# Factors associated with self-rated health in primary care in the South-Western health zone of Malawi

**DOI:** 10.1186/s12875-022-01686-y

**Published:** 2022-04-19

**Authors:** Stephen Kasenda, Eivind Meland, Øystein Hetlevik, Thomas Mildestvedt, Luckson Dullie

**Affiliations:** 1 Malawi Epidemiology and Intervention Research Unit, Lilongwe, Malawi; 2 Department of Global Public Health and Primary Care, University of Bergen, 5020 Bergen, Norway; 3 Partners in health, Neno, Malawi

**Keywords:** Malawi, Primary health care, Social determinants of health, Health status, Health care outcome assessment

## Abstract

**Background:**

Self-rated health (SRH) is a single-item measure of current health, which is often used in community surveys and has been associated with various objective health outcomes. The prevalence and factors associated with SRH in Sub-Saharan Africa remain largely unknown. This study sought to investigate: (1) the prevalence of poor SRH, (2) possible associations between SRH, and socio-demographic and clinical parameters, and (3) associations between SRH and the patients’ assessment of the quality of primary care.

**Methods:**

A cross-sectional study was conducted in 12 primary care facilities in Blantyre, Neno, and Thyolo districts of Malawi among 962 participants who sought care in these facilities. An interviewer-administered questionnaire containing the Malawian primary care assessment tool, and questions on socio-demographic characteristics and self-rated health was used for data collection. Descriptive statistics were used to determine the distribution of variables of interest and binary logistic regression was used to determine factors associated with poor SRH.

**Results:**

Poor SRH was associated with female sex, increasing age, decreasing education, frequent health care attendance, and with reported disability. Patients content with the service provided and who reported higher scores of relational continuity from their health care providers reported better SRH as compared with others.

**Conclusion:**

This study reports findings from a context where SRH is scarcely examined. The prevalence of poor SRH in Malawi is in line with findings from clinical populations in other countries. The associations between poor SRH and socio-demographic factors are also known from other populations. SRH might be improved by emphasizing continuity of care in primary care services.

## Introduction

Self-rated health (SRH) is a single-item measure of current health, which involves asking individuals to rate their health on a Likert scale or in comparison with their peers [1, 2]. It is often used as a measure of health in community surveys due to its simplicity and inclusive nature for various health determinants [2]. There is evidence that poor SRH is associated with increased: levels of inflammatory markers, need for healthcare service utilization, and risk of poor clinical outcomes [3–7]. However, SRH is non-specific and not diagnostic of any specific pathology. Hence, we need to investigate specific factors associated with SRH in various geographic and demographic settings. Various studies from the global north have also demonstrated that SRH is a stable concept that is formulated as early as adolescence and is affected by various factors including stress, various socio-demographic parameters, and social determinants of health [8–12]**.** Somatic symptoms, sleep deprivation, and unsatisfied relational needs have also been associated with poor SRH [13, 14].

High-quality primary care has been associated with good health outcomes such as better SRH, lower mortality, lower incidence of low birth weight, decreased need for hospital visitations, and generally better health at lower costs for primary care sensitive conditions [15–18]. The Primary Care Assessment Tool (PCAT) is a multi-dimensional tool for determining a patient’s assessment of the quality of primary care in various settings [19]. This instrument has been adapted and validated for use in Malawi [20]**.**

Although the association between the quality of primary care as assessed by PCAT, and SRH has been demonstrated by several studies, the association between different PCAT domains (e.g. comprehensiveness, coordination, family centeredness, and accessibility of care) and SRH are varying. For example one study, using the validated Tibet Primary Care Assessment Tool (PCAT-T), reported the total primary care score and individual domain scores as having associations with SRH whereas another study using the Korean Primary Care Assessment Tool (K-PCAT) found that only the total primary care score was associated with SRH status. [15, 18]. This phenomenon is probably due, at least in part, to variations in the study settings and the designs of tools validated for use in those settings.

In sub-Saharan Africa (SSA), where satisfaction with health care is lowest (42.4%), and good SRH is second-lowest (75.5%) after the former Soviet Union (69.6%) [21]**,** there is high variability in published findings from community-based studies on the prevalence of poor SRH in SSA [12, 22]. This phenomenon was even observed in one multi-national study that used uniform methods [21]**. **The factors associated with poor SRH in these studies are also different besides advancing age and female sex, and there is a paucity of clinical data on this topic [12, 22, 23].

This study sought to investigate: (1) the prevalence of poor SRH in the south-western health zone of Malawi, (2) possible associations between SRH, and various socio-demographic and clinical parameters, and (3) possible associations between SRH and the performance of the core quality dimensions of primary care.

## Methods

### Study setting and study population

Data were collected from patients who sought health care services in 12 health government-owned facilities located in three districts of the south-western health zone of Malawi, namely: Blantyre, Neno, and Thyolo. Blantyre and Neno were purposively chosen due to their unique characteristics of having an urban population (Blantyre), and the highest per capita health spending in the south-western zone respectively (Neno). Thyolo was randomly selected from the remaining five districts in the zone, which in addition to Thyolo include; Nsanje, Chikhwawa, Mwanza, and Chiradzulu. All government-owned primary care clinics and hospitals in these districts are in the first and second levels of the three tier health system respectively, and they operate as walk-in clinics that offer a standard essential health package with additional secondary care services being offered at the hospitals to patients for whom such is necessary. All health services in government owned facilities are offered free for all without any formal consultation lists. Thus, there is no financial barrier to accessing care, and all conditions beyond the scope of primary care services need to be referred to hospitals that offer secondary or tertiary health care.

Neno is the smallest of the three districts with a total population of less than 138,000, and a population density of 89 people per square kilometre in contrast with Blantyre and Thyolo, which have populations and population densities over 700,000 and 400 people per square kilometres respectively [24]. Over three-quarters of residents over the age of five years in Thyolo (79%) and Neno (77%) have no formal educational qualifications in contrast to 48% in Blantyre, which also has the highest proportion of people with tertiary education qualifications (7%) in contrast to less than 1% in the other districts [24].

The study population comprised of all adults of at least 18 years of age from the catchment area of the respective facilities who primarily sought care at the facility for at least two years and had visited the facility at least three times. The participants also needed to be capable of independently giving informed consent and answering the questions in the study questionnaire. Potential participants were excluded from the study if they could not give informed consent or if they were in urgent need of medical care at the time of data collection. The participant selection procedure has been detailed elsewhere  [20]: briefly, each data collector daily interviewed 12 randomly selected patients recruited in outpatient primary care clinics throughout the study period. Participants were recruited at a constant pre-calculated rate determined by the expected duration of each interview, average primary care consultation time, and the number of patients already present at the clinic at the beginning of the day shift. This approach ensured that none of the study participants got inconvenienced by their participation in the study.

### Data collection and analysis

#### Data collection

Data was collected through face-to-face interviews using the Malawi Primary Care Assessment Tool (PCAT-MW), which was validated for use in Malawi [20, 25]. The questionnaire has 29 items, which measure patients’ experiences of the five core dimensions of primary care: first-contact-access; continuity- communication; continuity-relation; comprehensiveness of services; coordination of care; and community orientation [25]. Five-point Likert scales were used to rate each item. Item scores on the Likert scale are allocated as follows: 1 indicating “definitely not,” 2 indicating “probably not,” 2.5 indicating “not sure” 3 representing “probably,” and 4 representing “definitely”.

#### Study measures

SRH was the main outcome variable of interest, and six level reporting of SRH outcomes were re-coded into a binary variable of either good (“Excellent”, “Very good” and “good”) or poor (“Fair”, “poor” and “very poor”) SRH. To ensure that the participants’ consideration of health would be more holistic and consistent with the World Health Organization definition of health, the word “umoyo” in the Chichewa vernacular was used in the study questionnaire when referring to health. Umoyo is used in the vernacular to refer to health in a broad sense that includes consideration of mental and social wellbeing unlike its somato-centric alternative “Thanzi”.

For purposes of this study, the PCAT-MW was augmented with interview questions on SRH and socio-demographic characteristics (sex, age, employment status, level of education attained) of each participant. Other variables were: duration of health facility affiliation, estimated time travelled to the health care facility, frequency of clinic visits within the immediate past 24 months, and the presence of disabilities.

Each primary care domain score was derived by dividing the total domain score by the number of items in the domain. Main domain scores were graded according to the standard Likert scale of the study with scores ≥ 3 being considered ‘acceptable to good performance’ and scores < 3 as ‘poor performance’. The total primary care score was calculated as the sum of all mean domain scores.

#### Statistical analysis

Descriptive statistics were performed to determine the prevalence of poor SRH and the socio-demographic parameters of the study participants. The proportions of socio-demographic data by SRH status were analysed using the Pearson chi-square test. Crude associations between variables of interest and poor SRH, and all variables with a crude odds ratio *p*-value of 0.1 or less were entered in a binary logistic regression model with poor SRH as the dependent variable of interest. A 5% significance level was used for all inferential statistics.

## Results

### Distribution of socio-demographic and healthcare variables

As shown in Table 1, 1001 eligible people were approached, and 962 (96.1%) were recruited into the study. Most of the participants were female (64.0%; *n* = 616), aged between 18 and 45 years (82.1%; *n* = 790), or residents of a rural area (68.7%; *n* = 661). Eighty-eight percent (*n* = 854) of participants had attained some formal education, with 52.8% (*n* = 508) having attained primary school education only, and 5.2% (*n* = 50) having some tertiary education. Thus, the overall educational status and age distribution of the participants approximated published findings from other community-based studies in Malawi and our estimated general distribution of the 2018 population census figures. [24, 26]. Disabilities were self-reported among 11.6% (*n* = 112) participants only. Most participants sought care at a primary care clinic (70.6%; *n* = 679), had a primary affiliation to the study facility (94.8%; *n* = 912), had been affiliated with the same facility for at least four years (72.8%; *n* = 701), had sought care at a health facility more than 5 times in the immediate past 24 months (549; 57.1%).

**Table 1:** Distribution of participant and healthcare characteristics, and their association with poor self-rated health status

	**Distribution of self-rated health status**				**Unadjusted Odds of poor SRH**		**Adjusted Odds of poor SRH**
**Characteristic**	**Total;** ***N* = 962 (%)**	**Good SRH; *n* = 621 (%)**^a^	**Poor SRH; *n* = 341 (%)**^a^	**χ2** ***P*-value**	**Unadjusted Odds Ratio (95% CI)**	***P*-value**	**Adjusted Odds Ratio** **95% CI)**
**Study site**							
Blantyre	301 (31.3)	205 (68.1)	96 (31.9)	0.005	0.99 (0.71—1.37)	0.005	1.35 (0.86—2.11)
Neno	303 (31.5)	173 (57.1)	130 (42.9)		1.59 (1.16—2.18)		1.61 (1.08—2.38)
Thyolo	358 (37.2)	243 (67.9)	115 (32.1)		1 (ref)		1 (ref)
**Sex**							
Female	616 (64.0)	377 (61.2)	239(38.8)	0.004	1 (ref)	0.004	1.64 (1.21—2.23)
Male	346 (36.0)	244(70.5)	102(29.5)		1.52 (1.14—2.01)		1 (ref)
**Age**							
18- 30 years	448 (46.6)	323 (72.1)	125 (27.9)	< 0.001	1 (ref)	< 0.001	1 (ref)
31—45 years	342 (35.6)	212 (62.0)	130 (38.0)		1.58 (1.17—2.14)		1.4 (1.01—1.93)
≥ 46 years	128 (13.3)	86 (50.0)	86 (50.0)		2.58 (1.8—3.72)		2.2 (1.45—3.33)
**Highest attained education**							
None	108 (11.2)	44 (40.7)	64 (59.3)	< 0.001	3.63 (2.32—5.69)	< 0.001	2.51 (1.5—4.2)
< 5 years primary	206 (21.4)	120 (58.3)	86 (41.8)		1.79 (1.25—2.57)		1.6 (1.06—2.42)
5 – 8 years primary	302 (31.4)	210 (69.5)	92 (30.5)		1.09 (0.78—1.53)		1.07 (0.74—1.55)
Secondary education or more	346 (36.0)	247 (71.4)	99 (28.6)		1 (ref)		1 (ref)
**Employment**							
Part-time or full time job	273 (28.4)	179 (65.6)	94 (34.4)	0.352	1.03 (0.75—1.43)	0.354	
Self-employed	395 (41.1)	262 (66.3)	133 (33.7)		1 (ref)		
Homemaker	294 (30.5)	180 (61.2)	114 (38.8)		1.25 (0.91—1.71)		
**Duration of facility affiliation**							
6 months to 2 years	153 (15.9)	102 (66.7)	51 (33.3)	0.705	0.88 (0.61—1.27)	0.704	
2 – 4 years	107 (11.1)	72 (66.7)	36 (33.3)		0.88 (0.57—1.35)		
> 4 years	701 (73.0)	447 (63.7)	255 (36.2)		1 (ref)		
**Frequency of clinic visits in 2 years**							
3–5 times	413 (42.9)	293(70.9)	120(29.1)	< 0.001	1 (ref)	< 0.001	1 (ref)
> 5times	549 (57.1)	328 (59.7)	221(40.3)		1.65 (1.25—2.16)		1.57 (1.17—2.11)
**Travel time to facility**							
< 30 min	316 (32.8)	213 (67.41)	103 (32.59)	0.345	0.79 (0.58—1.08)	0.344	
30-60 min	247 (25.7)	160 (64.78)	87 (35.22)		0.89 (0.64—1.24)		
> 60 min	399 (42.5)	248 (62.16)	151 (37.84)		1 (ref)		
**Disabled**							
No	850 (88.4)	566(66.6)	284(33.4)	< 0.001	1 (ref)	< 0.001	1 (ref)
Yes	112 (11.6)	55(49.1)	57 (50.9)		2.07 (1.39—3.07)		2.14 (1.39—3.3)
**Perceived quality of treatment received**							
Poor	466 (48.4)	279 (59.9)	187 (40.1)	0.003	1.18 (1.01—1.37)	0.003	1.68 (1.26—2.26)
good	496 (51.6)	342 (69.0)	154 (31.1)		0.99 (0.84—1.17)		1 (ref)
**Type of health facility**							
Primary care clinic	679 (70.6)	460 (67.8)	219 (32.3)	0.001	1.24 (1.05—1.45)	0.002	1 (ref)
Hospitals offering primary care services	283 (29.4)	161 (56.9)	122 (43.1)		0.91 (0.8—1.04)		1.25 (0.78—1.99)
**PCAT-MW dimensions**							
	**Total;** ***N* = 962 (%)**	**Good SRH;** ***n* = 621 (95%CI)**	**Poor SRH; *n* = 341 (95%CI)**	**T test** ***p*-value**	**Unadjusted Odds Ratio (95% CI)**	***P*-value**	**Adjusted Odds Ratio** **(95% CI)**
First-contact access	962 (100)	2.54 (2.47—2.61)	2.66 (2.56—2.75)	0.04	1.18 (1.01—1.37)	0.04	0.99 (0.81—1.22)
continuity-communication	962 (100)	3.47 (3.41—3.53)	3.46 (3.38—3.54)	0.884	0.99 (0.84—1.17)	0.884	
continuity- relationship	962 (100)	1.88 (1.82—1.95)	1.78 (1.69—1.87)	0.086	0.87 (0.75—1.02)	0.086	0.82 (0.68—0.98)
comprehensiveness of services available	962 (100)	2.23 (2.17—2.30)	2.38 (2.30—2.47)	0.009	1.24 (1.05—1.45)	0.009	1.12 (0.87—1.43)
comprehensiveness of services provided	962 (100)	2.56 (2.48—2.64)	2.47 (2.36—2.58)	0.172	0.91 (0.8—1.04)	0.172	
community orientation	962 (100)	2.90 (2.82—2.97)	2.87 (2.77—2.97)	0.641	0.97 (0.84—1.11)	0.641	
coordination of care	962 (100)	1.95 (1.74—2.15)	2.22(1.90—2.53)	0.146	1.16 (0.95—1.41)	0.146	

^a^mean scores and their 95% confidence intervals are presented here

Neno district had the highest proportion of participants who, (I) had more than five primary care visits in the immediate past 24 months 64.0%; versus 61.5% & 44.9% in Thyolo & Blantyre respectively; χ^2^ *p*-value < 0.001), (II) perceived the treatment they received to be of good quality (60.7% versus 52.2% & 41.5%; χ^2^ *p*-value < 0.001), and (III) had to travel for more than an hour to access primary care (60.1% versus 46.7% & 16.6%; χ^2^ *p*-value < 0.001).

### Prevalence of poor self-rated health

Three hundred and forty-one participants (35.5%) reported having poor SRH. Statistically significant higher prevalence of poor SRH was present among participants who; were female (38.8% versus 29.5%; *p* = 0.004), disabled (50.9% versus 33.4%; *p* < 0.001), sought care at clinics as compared to hospitals that offer primary care (43.1% versus 32.3%; *p* = 0.001) or had more than five primary care visits in the immediate past 24 months (40.3% versus 29.1%; *p* < 0.001). Participants who perceived the treatment provided at the index facility to be of poor quality were also more likely to have poor SRH (40.1% versus 31.0%; *p* = 0.003).

Prevalence of poor SRH increased with age such that 50% (95% CI: 42.3—57.7) of participants over the age of 45 had poor SRH compared to 38.0% (95% CI: 32.8—43.4) and 27.9% (95% CI: 23.8—32.3) among participants aged 31–45 years and 18–30 years respectively. Poor SRH, however, exhibited an inverse relationship with the participants’ highest attained education such that 59.3% (95% CI: 49.4—68.6) of participants with no formal education had poor SRH compared to 28.6% among those who had attained secondary or tertiary education (Table 1).

### PCAT-MW scores and self-rated health status

The mean total primary care score was 16.1 (95% CI: 15.87—16.33), and scores suggestive of good quality care (scores ≥ 21) were computed from 10% (*n* = 96) of the participants only. There was no statistically significant difference in the overall scores computed across the SRH divide (*p*-value = 0.84).

### PCAT-MW factors and socio-demographic factors associated with poor SRH

Table 1 demonstrates how demographic characteristics and level of perceived quality of health care related to SRH in unadjusted bivariate analyses. Poor SRH varied significantly between the different districts, Sex, age of participants, level of highest attained education, and presence of disability. Higher odds of poor SRH were also recorded for the following categories of binary variables: (I) those who visited hospitals (OR: 1.59; *p*-value = 0.002), (II) those who received treatment they perceived to be of poor quality (OR: 1.49, *p*-value = 0.003), and (III) those who sought care more than 5 times within the immediate past 24 months (OR: 1.65; *p*-value < 0.001). Participants rating increased level of first contact access, and comprehensiveness of services available also had statistically significant positive associations with the odds of poor SRH.

**Fig. 1:**
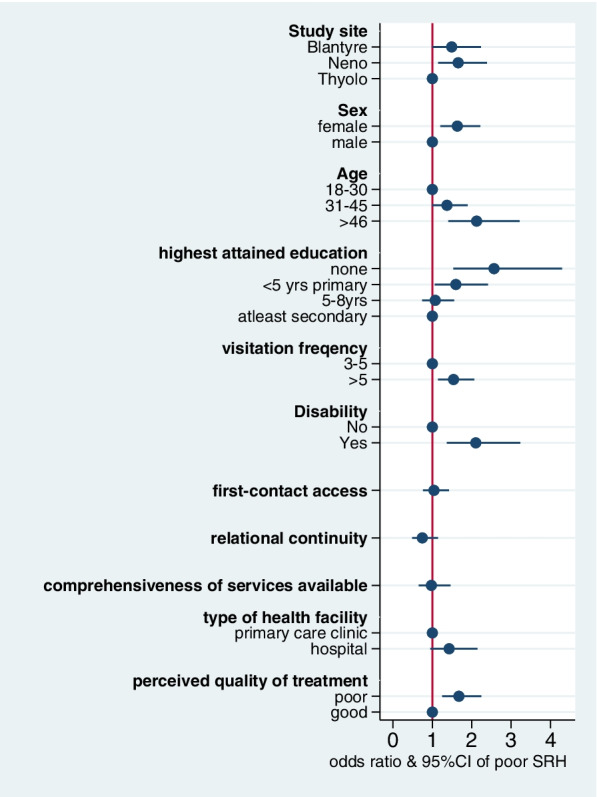
Adjusted odds ratios and 95%CI of factors associated with poor SRH

In the fully adjusted model (Table 1 and Fig. 1), participants who were from Neno district (OR: 1.61; 95% CI: 1.08—2.38), female (OR: 1.64; 95% CI: 1.21—2.23), or with disabilities (OR: 2.14; 95% CI: 1.39—3.3) had statistically significant higher odds of poor SRH than their counterparts. The odds of poor SRH increased with age with the highest odds of poor SRH being recorded in the oldest group (OR: 2.20; 95% CI: 1.45—3.33). Poor SRH also had an inverse relationship with the education of participants, such that the odds of poor SRH were highest among those with no formal education (OR: 2.51; 95% CI: 1.5—4.2). Participants who sought care more than five times in the immediate past 24 months had 1.57 higher odds of poor SRH (95% CI: 1.17—2.11) whereas those who received treatment they perceived as poor had 1.68 higher odds of poor SRH (95% CI: 1.26—2.26). Relational continuity was the only PCAT-MW dimension that had a statistically significant inverse association with poor SRH in the adjusted model with the odds of poor SRH decreasing by a factor of 0.82 (95% CI: 0.68—0.98) for every unit increase in the domain score.

## Discussion

In this survey of primary care patients in the south-western health zone of Malawi, one-third of patients had poor SRH. Poor SRH was associated with female sex, increasing age, decreasing education, frequent HC attendance, and reported disability. Patients content with the service provided and who reported high scores of relational continuity from their health care providers reported better SRH as compared with others.

### Prevalence of poor SRH

To the best of our knowledge, this is the first study on self-rated health in the south-western health zone of Malawi. Although the prevalence of poor SRH is lower than those of other Sub-Saharan African (SSA) countries with higher per capita expenditure on health, and comparable to findings from high-income settings it ought to be considered as suboptimal and a cause for concern [12–14, 22, 23, 27]. The latter is due to Malawi’s good SRH prevalence that is below the continental mean [21]**.** Resource constraints, growing double burden of communicable and non-communicable diseases, increasing life expectancy, and declining mortality from conditions that once carried high mortality also portend further declines in prevalence of good SRH [26, 28]**.** Since SRH is a stable construct, increasing life expectancy and declining HIV-associated mortality also portend a higher burden of poor SRH [29, 30]**.**

The prevalence of poor SRH also highlights the importance of looking beyond the provision of freely accessible care, as is the case in Malawi, but also good quality patient-centred care to improve clinical outcomes and patients’ confidence in the health system [31].

### SRH & socio-demographic characteristics

The pattern of disparity in SRH outcomes across the study sites was consistent with findings from other studies, which report better SRH among urban residents and participants with a better socioeconomic context [22, 23, 32, 33]. Contrary to our expectations, the odds of poor SRH were highest in Neno despite Neno having the highest per capita expenditure on health, the highest proportion of participants who considered the treatment they received to be of good quality and being anecdotally reported as a popular health tourism destination for residents from neighbouring districts. Neno also has integrated care programmes, which are unique to the district, and are credited to have resulted in increased case finding and uptake of various primary care services including chronic infectious & non-infectious diseases, with consequential improvements in survival rates [34–36]**.**

This SRH pattern suggests that the aforementioned qualities of primary care in Neno, though necessary, might be insufficient in efforts to improve SRH trajectories of communities such as in Neno. The pattern is, however, consistent with the “paradox of health” observed by Barsky, where excellent health, in the presence of (I) advanced medical care, (II) heightened consciousness of health, (III) commercialization of health, and (IV) medicalization of daily life, is associated with poor subjective health [37]. The relative importance of each of the elements in this complex web of factors in Malawi is not yet known, but these elements highlight the overall importance of patients’ social context in their overall health and SRH trajectories. However, Barsky points at a paradoxical relationship that may be universal: health care succeeding at combating disease and mortality will not necessarily improve the perceptions of health of patients and populations.

The finding of female sex as an independent factor associated with poor SRH, though consistent with literature from other settings, is counterintuitive because (I) Malawi provides free health care for all, and (II) men in Malawi have a greater burden of disease and lower life expectancy [32, 38, 39]**.** On the other hand, SRH is often associated with social, contextual factors and subjective wellbeing [30]**.** This disparity, which has also been demonstrated among adolescents in other studies, can be attributed, at least in part to the male–female health survival paradox that may result from community views on health and masculinity [40–43]. These result in women having more contact with the health system, having more diagnoses of conditions that are often non-lethal, and potentially having more knowledge about their health [39, 43]. Consequently, women may be more responsive to changes in their bodies and may, more often than men, factor these when asked to rate their health. Other studies have also attributed Sex disparities in SRH, at least in part, to differences in stress and social determinants of health [38, 40].

### SRH & quality of primary care

Relational continuity is independently associated with a reduced cost of care, improvements in uptake of preventive care, adherence to treatment, patient satisfaction with care, and health outcomes [44]. It is a surrogate marker of patients’ trust and satisfaction, and its importance is expected to increase as the burden of chronic diseases grows [44, 45]**.** Relational continuity is not an innate aspect of Malawi’s approach to primary care. Based on the personal experience of the authors (SK and LD), relational continuity in Malawian public primary care facilities is often a consequence of patient preference, healthcare worker shortages, and the presence of chronic diseases (e.g. HIV and diabetes). The presence of statistically significant associations between relational continuity and better SRH, albeit without systemic efforts to institute the same, suggests that relational continuity is a crucial and cost-effective ingredient for improving the quality of primary care and SRH in the study communities.

Participants’ age, presence of disabilities, and highest attained education are markers of social determinants of health. Marginalised populations (e.g. the disabled and people of low socioeconomic status) and the elderly are likely to be the greatest victims of these factors since they tend to experience socio-economic exclusion, reduced access to care, and numerous unique healthcare needs. Thus, the pillars of universal health coverage seem indispensable in the quest to improve SRH. The highest attained education of a participant is a valid measure of socioeconomic status and is probably associated with health knowledge and perception of self-efficacy [46].

The absence of a statistically significant association between comprehensiveness of services and SRH was another unexpected finding, which is probably a consequence of the uniformity in the range of services provided as part of the essential health package in Malawian primary care facilities.

### Limitations and strengths

Our study has several limitations. The most important is the cross-sectional design where we cannot establish causal relationships between any of the factors identified and SRH. Another limitation is in the exploration of patient’s subjective reports. However, procedural strategies, namely: (I) explaining the purpose of the study to participants, (II) use of unambiguous Likert scale labels, and (III) using a mixture of response approaches, were used to minimise recall and desirability tendencies on the part of participants. All questions were asked in the vernacular language with clear wording that is consistent with similar international tools.

Although the tool is validated for use in our setting, the use of the tool in a clinical setting may have made some of the PCAT domains, especially “first-contact access”, prone to selection bias. Data on the objective health status of the participants, except self-reported disability, was not collected. Thus, the effect of various pathological processes, especially mental health conditions, which may affect one's perception of the quality of care received, were not factored in the analysis. We also recognise the existence of the possibility that the findings we have attributed to the health paradox may be due to confounders not adjusted for in this study.

## Conclusion

This study reports findings from a context where SRH is scarcely examined. The prevalence of poor SRH in Malawi is in line with findings from clinical populations in other countries. The associations between poor SRH and lower socioeconomic status are also known from other populations. SRH might be improved by emphasizing continuity of care in primary care services.

## Data Availability

The datasets generated and analysed during the current study are not publicly available due to lack of prior approval from the NHSRC but are available from the corresponding author on reasonable request, subject to approval from NHSRC.

## Abbreviations

PCAT: Primary Care Assessment Tool
PCAT-MW: Malawi Primary Care Assessment Tool
SRH: Self-rated health
SSA: Sub-Saharan Africa
